# Inferring the rules of social interaction in migrating caribou

**DOI:** 10.1098/rstb.2017.0385

**Published:** 2018-03-26

**Authors:** Colin J. Torney, Myles Lamont, Leon Debell, Ryan J. Angohiatok, Lisa-Marie Leclerc, Andrew M. Berdahl

**Affiliations:** 1 School of Mathematics and Statistics, University of Glasgow, Glasgow G12 8QW, UK; 2 Centre for Mathematics & the Environment, University of Exeter, Penryn TR10 9EZ, UK; 3 TerraFauna Wildlife Consulting, 19313 Zero Avenue, Surrey, BC, Canada, V3Z 9R9; 4 Government of Nunavut, Department of Environment, Kugluktuk, NU, Canada, X0B 0E0; 5 10 Aniakvik Road, Cambridge Bay, NU, Canada, X0B 0C0; 6 Santa Fe Institute, Santa Fe, NM 87501, USA; 7 School of Aquatic and Fishery Sciences, University of Washington, Seattle, WA 98195, USA

**Keywords:** barren-ground caribou (*Rangifer tarandus groenlandicus × pearyi*), collective behaviour, social interaction rules, migration modelling, unmanned aerial vehicles (UAV)

## Abstract

Social interactions are a significant factor that influence the decision-making of species ranging from humans to bacteria. In the context of animal migration, social interactions may lead to improved decision-making, greater ability to respond to environmental cues, and the cultural transmission of optimal routes. Despite their significance, the precise nature of social interactions in migrating species remains largely unknown. Here we deploy unmanned aerial systems to collect aerial footage of caribou as they undertake their migration from Victoria Island to mainland Canada. Through a Bayesian analysis of trajectories we reveal the fine-scale interaction rules of migrating caribou and show they are attracted to one another and copy directional choices of neighbours, but do not interact through clearly defined metric or topological interaction ranges. By explicitly considering the role of social information on movement decisions we construct a map of near neighbour influence that quantifies the nature of information flow in these herds. These results will inform more realistic, mechanism-based models of migration in caribou and other social ungulates, leading to better predictions of spatial use patterns and responses to changing environmental conditions. Moreover, we anticipate that the protocol we developed here will be broadly applicable to study social behaviour in a wide range of migratory and non-migratory taxa.

This article is part of the theme issue ‘Collective movement ecology’.

## 1. Introduction

Migrating species play a keystone role in the functioning of many ecosystems; they transport nutrients, connect disparate communities and act as both major resource consumers and prey for resident species [1]. Recent technological developments have led to an unprecedented insight into the movement patterns of animals [2]. However, despite the fact that many species migrate in groups [3,4], most studies of animal migration neglect the potential role of social interactions on movement decisions. Much research has shown that interactions are important to consider, both in the context of collective animal behaviour [4–8] and more generally in the study of complex systems [9].

While the potential importance of interactions in the decision-making of animal groups is recognized, the barrier to quantifying these interactions among wild, free-ranging animals is the difficulty in obtaining simultaneous, fine-scale trajectories for every animal in a group [10]. Such trajectories are more easily obtained for small-bodied animals in a laboratory setting, and have been used to infer rules of social interactions in several species [11–13]. Video footage has also been used to analyse interaction rules in the field [14–16] but these studies are restricted to non-migratory situations, where foraging or predator evasion, rather than navigation or large-scale movement are the primary motivation.

Increasingly, GPS (Global Positioning System) collars have been used to obtain simultaneous trajectories of multiple individuals within a group [17]. For some species that form cohesive and stable groups, all individuals have been tracked [18] including for migratory species such as bald ibis (*Geronticus eremita*) [19]. In other cases it has been possible to track the majority of individuals in the group. Crofoot *et al.* [20] were able to capture, tag and track over 80% of the adult members of a baboon troop (*Papio anubis*) and obtained high temporal resolution movement data over a two week period [21–24]. For species that form dynamic groups, or those where it is only possible to track smaller proportions of the group, social interactions have still been investigated using GPS data [25]. Several studies have examined the relationship between individuals and group average headings or centroids [26,27] revealing how individuals respond to collective properties of the group. Pairwise interactions have also been analysed using GPS collars [28] and passive integrated transponder (PIT) tags [29] to infer the structure of social networks underlying movement data.

While GPS collars are able to collect the high frequency data required to detect and differentiate between interaction rules [23,24,26], there are limitations. The proportion of individuals within a group that can be tracked is strongly dependent on the size and stability of groups, and the logistics of capturing and tagging animals. Recent advances in unmanned aerial systems (UAS) [30–33] and automated computer vision [34,35] offer a complementary technology to the use of individual telemetry with the potential to deliver the simultaneous trajectories needed to infer interaction rules of wild populations in a variety of settings [10]. In this work, we applied these technologies to understand how social interactions among conspecifics influence the movement decisions of a free-ranging migratory ungulate in the Canadian Arctic.

Barren-ground caribou (*Rangifer tarandus groenlandicus*) are highly social [36] and make some of the longest terrestrial migrations on the planet [37]. The purpose of these migrations is to travel from more southerly wintering grounds to discrete, population-specific, high-latitude calving grounds. Herding behaviour is important during migration, because the locations of, and routes to, these calving sites are passed down through social learning [38]. Moreover, dispersal between these sites appears to be socially mediated, through fission–fusion dynamics on the wintering grounds [39]. Additionally, recent research suggests that social interactions influence directional decisions during migration [27]. However, the precise nature of those social interactions for caribou remains unclear.

Dolphin and Union caribou are genetically different from other barren-ground caribou (*Rangifer tarandus groenlandicus*) and from Peary caribou (*Rangifer tarandus pearyi*) [40,41] and display distinct behaviours. Although adopting an individualistic calving strategy like the Peary caribou, they aggregate in numbers on the southern coast of Victoria Island, Nunavut, as sea-ice forms, before crossing the ice to continue their fall migration to their wintering grounds on the Canadian mainland [42,43].

We developed a UAS-based approach to study *in situ* the collective movement behaviour of Dolphin and Union caribou. Filming was undertaken in November 2015 as the caribou approached the coast of Victoria Island prior to crossing to the mainland (see electronic supplementary material for details). Footage was obtained using a commercially available unmanned aerial system, the 3D Robotics Solo, and processed using the open source computer vision package OpenCV. Once tracked the footage resulted in 12 h 40 min worth of individual tracks, with an average track length of 59 s and a maximum length of 9 min. Herds consisted of up to 51 individuals with an average herd size of 15.26. We use a Bayesian approach to infer interaction rules [13,,44,45] for groups of migrating caribou and predictive information criteria [46,47] to determine the most parsimonious mathematical description of these interactions.

**Figure 1.**
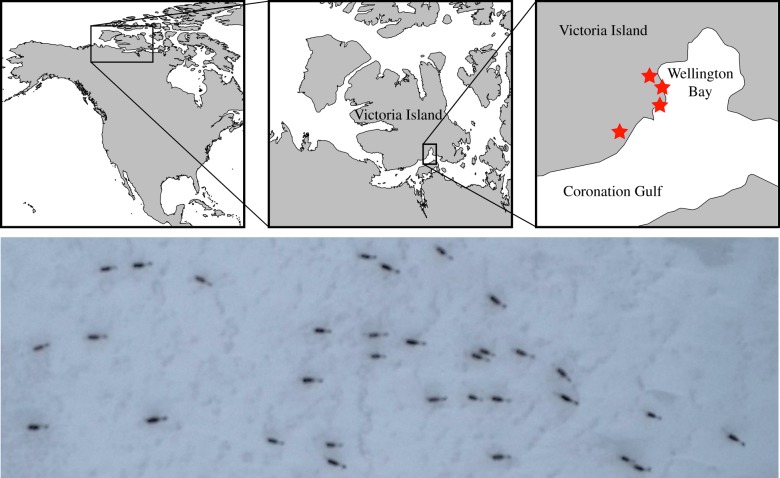
Study system. (*a*) Map of study area on Victoria Island, Canada. Red stars indicate location of (multiple) UAV flights. (*b*) Portion of a still from UAV-collected video footage of caribou herd.

**Figure 2.**
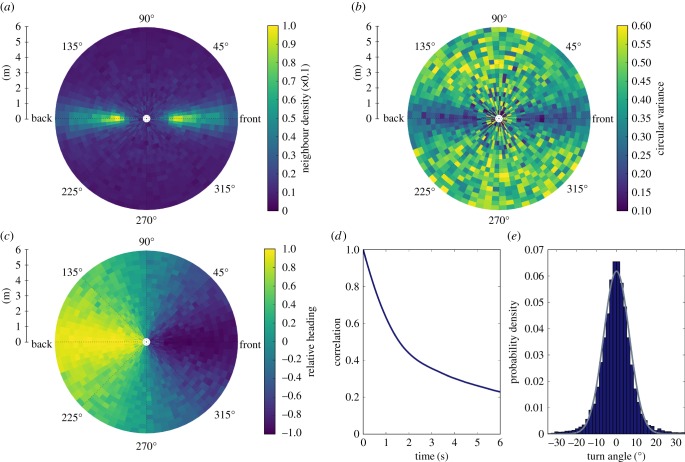
Relative positions and velocities. (*a*) Heatmap of relative positions. Values indicate the probability a neighbour is located in that position relative to a focal individual. (*b*) Variance in average heading. Values show the circular variance of the headings of individuals at each location. (*c*) Relative orientation. The average heading of individuals within each cell is projected onto the vector pointing toward the focal individual. The length of the resultant vector is shown. Positive (negative) values indicate movement towards (away from) the focal individual. (*d*) Autocorrelation in movement heading as a function of time. (*e*) Probability of movement step heading change within a 2 s interval. Line shows a fitted Gaussian curve (*μ* = 0, *σ* = 6.54).

A still image taken from the obtained video footage is shown in figure 1. From the tracked data positions and headings of all individuals were recorded. Heat maps of relative positions and orientations of neighbours are shown in figure 2. These heat maps emphasize the clear tendency of caribou herds to form lines as they migrate. Figure 2*a* shows that neighbour density is centred front and back of an individual at a distance of approximately 2 m. The lines caribou form tend to be well-aligned with lower variance in heading along the front-back axis (figure 2*b*). Although there is higher variation in the headings of neighbours to the left and right of individual caribou, figure 2*c* shows that herds display consistent aligned motion.

From continuous trajectories properties of caribou movement were calculated. Figure 2*d* shows the autocorrelation in movement direction for all individuals. A change in the rate of decay in correlation is apparent after 2 s so we select this interval as the time scale for our analysis; however, our results were not sensitive to this particular value (see electronic supplementary material, figure S4).

## 2. Movement model

To analyze movement decisions we employ a discrete-time continuous-space biased random walk model. Based on the autocorrelation of the continuous trajectories we discretize trajectories into movement steps of 2 s duration then, following the approach of McClintock *et al.* [48], each movement step is modelled as a random draw from a wrapped Cauchy distribution centred on an expected heading. The probability the movement step at time *t* is in the direction *θ*_*t*_ is given by2.1
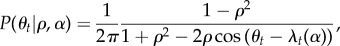
where λ_*t*_ is the expected heading defined as a function of model parameters *α*, and *ρ* determines the variance around this heading, i.e. the amount of unpredictability of the movement process.

The expected heading for each step reflects a tendency for movement to be biased by a variety of factors, including environmental features, the positions of conspecifics and the persistence of individual motion. Within the framework proposed by McClintock *et al.* [48] various drivers of movement may be incorporated into the model by taking λ_*t*_ to be a weighted average of the heading indicated by each potential influence.

Given a model of λ_*t*_, equation (2.1) allows us to obtain a likelihood function for the parameters of the model and the predictability of movement *ρ* given this model (see electronic supplementary material for further details). From this likelihood function, we may compare the performance of various models of interaction and employ computational Bayesian methods using the PyMC package [49] to obtain posterior distributions of parameter values conditional on the observed data.

In total, we compare eight models that incorporate three potential drivers of movement decisions: directional persistence, environmental features and social cues. The expected heading used within equation (2.1) is a weighted average of the headings associated with each of these factors,2.2
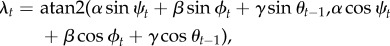
where *α* + *β* + *γ* = 1, *ψ*_*t*_ is the heading dictated by social cues, *ϕ*_*t*_ is an estimate of environmental forces, and *θ*_*t*−1_ is the previous heading.

By taking *α* = *β* = 0 we obtain the simplest model that assumes movement follows a correlated random walk [50]. Here the only predictor of step direction is the heading at the previous time step. If *β* > 0 then a model that incorporates the features of the environment, such as trails or obstacles, is obtained. As the true nature of these features is unknown, we estimate these features as in Dalziel *et al.* [27] by examining the average heading of all individuals within the herd at each fixed point in space.

To incorporate the effects of social cues on movement decisions and create a socially informed correlated random walk model [51], we incorporate a social heading into the model that is a function of the headings and positions of near neighbours. We evaluate the performance of three forms of interactions: a metric interaction zone where individuals are influenced equally by all neighbours within a fixed range, a topological interaction model where the nearest *K* neighbours affect decisions, and a model where influence decays exponentially with distance, akin to the local crowded horizon model proposed in Viscido *et al.* [52]. We assess all three sets of interaction rules, with and without alignment forces, meaning a total of six socially informed movement models are compared. The mathematical details of each of these models can be found in the electronic supplementary material along with a validation of the approach on individual-based simulations of interacting and non-interacting individuals responding to various external cues.

## 3. Model comparison

The different models described above were compared using two predictive information criteria, WAIC (widely applicable information criterion) [53] and DIC (deviance information criterion) [54]. Both these model comparison statistics make use of the posterior parameter distributions to estimate out-of-sample model fit. They are thus less reliant on the asymptotic assumptions of criteria based on maximum-likelihood estimates and have been shown to be effective tools in the analysis of collective movement data [55].

Relative scores and model rankings are shown in table 1 while posterior statistics from Markov chain Monte Carlo (MCMC) runs are shown for each model in the electronic supplementary material. The results in table 1 show clearly the influence of social interactions on the fine-scale movements of caribou. The random walk model, which is simply a correlated random walk, is the worst performing model and we take this as a baseline for model scores. A significant improvement is attained if we also make use of the average headings of the herd (excluding the focal individual) at each point in space. This environment model makes use of herd movements to estimate the environmental pathways that individuals follow but has no explicit inter-individual interactions.

**Table 1.** Model selection scores.

model	social	ΔWAIC	rank	ΔDIC	rank
exponential decay + alignment	Y	−3265	1	−3306	1
metric + alignment	Y	−3213	2	−3226	2
metric	Y	−3166	3	−3176	4
exponential decay	Y	−3160	4	−3187	3
topological + alignment	Y	−3031	5	−3050	5
topological	Y	−2933	6	−2959	6
environment	N	−1768	7	−1770	7
random walk	N	0	8	0	8

**Figure 3.**
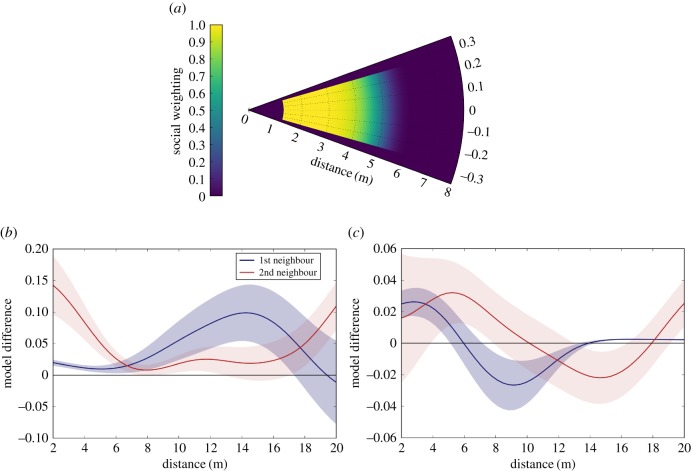
Inferred interaction rules. (*a*) Weighting given to neighbours as a function of their position relative to the focal individual for the best-fitting model. Parameters of social interaction model are taken from maximum *a posteriori* probability estimates. (*b*) Relative performance of exponential decay model versus topological model. We use the optimal parameters for each model then compare performance as a function of the distance to the nearest neighbour and the second nearest neighbour. Positive values indicate a movement step was more likely given interactions follow the decaying model than the topological model. Data is binned into 1 m bins, an average difference in probability for each bin is calculated, and the results are smoothed using a Gaussian filter. Shaded regions indicate the standard error. (*c*) Relative performance of exponential decay model versus metric model. As in (*b*), positive values indicate the decay model has higher relative performance.

By incorporating social behaviour, models are greatly improved, with all models that include direct social interaction outperforming the simpler models. The closest approximation to the real interactions between caribou is found using the exponentially decaying model with alignment. A plot of the weighting given to neighbours in the best-fitting model is shown in figure 3*a*. We note that both information criteria provide almost identical model rankings, with only the exponential decay and metric models without alignment showing inconsistent results.

All models with alignment forces display a better agreement with the data, showing that both attraction and copying directional choices play an important role in driving movement decisions, and this finding is independent of the exact model used to approximate interactions. In order to better understand the relative performance of the models, models are set at their maximum-likelihood parameter values and the difference in their performance as a function of the distance to the nearest and second nearest neighbours is shown in figure 3*b*–*c*. The average difference in the probability of an observed movement step given the exponential model and the topological model is shown in figure 3*b*, while the difference between the exponential model and the metric model is shown in figure 3*c*.

While the plots are smoothed and do not represent a formal analysis of the effect of distance, they do provide a heuristic insight into the reasons for the performance of the models. The topological model most closely matches the data when only a single neighbour exerts influence (as the optimal value of *K* = 1). Figure 3*b* shows that the topological model performs badly when this closest neighbour is far away, as the model assumes this individual exerts an influence when it is effectively out of range. The topological model also performs far worse than the metric model when the second closest neighbour is nearby. This reveals that the second neighbour is important and impacts decisions, although not with the same weight as the closest neighbour.

The difference in influence of neighbours is clear when comparing the metric model (all neighbours weighted equally) with the exponentially decaying model as shown in figure 3*c*. The decaying model performs better when both the first and second neighbours are close by as it provides less weighting to the information provided by the second individual. The metric model is better when the closest neighbour is further away. In this scenario the decaying model is optimized to accurately reflect the relative weighting between first and second neighbours, and performs badly when these neighbours are both further away.

## 4. Variation in social information use

Model comparisons were performed under the assumption that all individuals make use of social cues in the same manner. In reality this will not be true and we expect that age, sex, social status and reproductive status will affect the use of social cues. Variation in an individual's behaviour over time will also occur due to the changing motivations and pressures that drive caribou movement patterns throughout the year. Detecting variation in social information use between and within individuals will provide important insight into the leadership dynamics of these herds, and also help reveal the drivers of the migration.

Within the framework outlined above we are able to detect significant variation in the behaviour of individuals according to their life stage. We manually classified each caribou as either a calf, an adult (small bulls, cows and yearlings) or a large bull. Assignment was performed after trajectories were linked and the process involved iterating over all individual tracks, displaying a zoomed-in video of the individual in question, then manually annotating the track with a key to indicate its demographic class.

**Figure 4.**
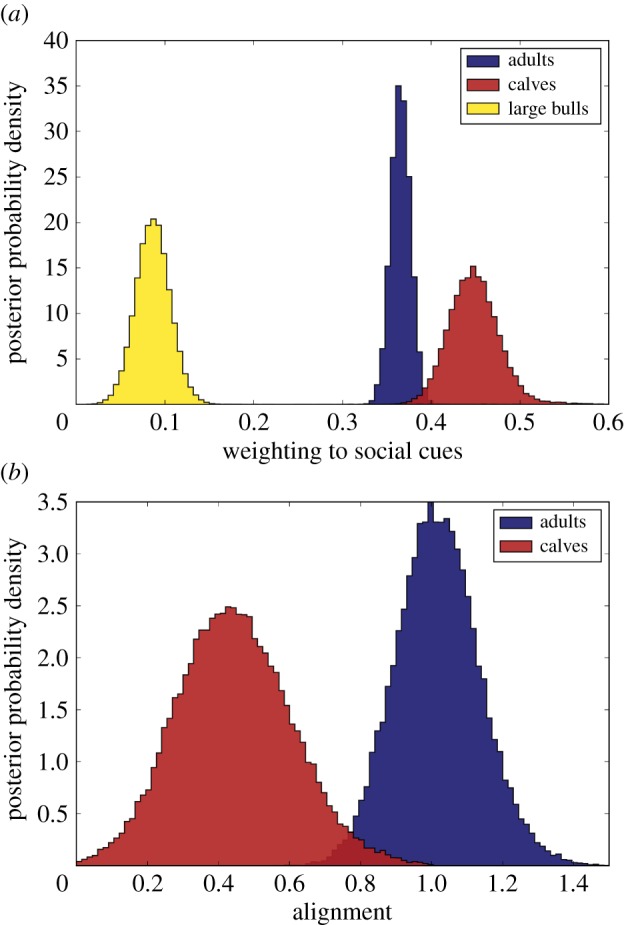
Variation in social information use. (*a*) Posterior distribution for weighting given to social cues, when individual caribou are classified as either calf, large bull or adult (small bulls, cows and yearlings). (*b*) Comparison of the posterior distribution of the alignment strength for adult caribou (not including large bulls) and calves. Parameters are shown for the exponential decay model. Clear variation is shown, with calves displaying greater tendency for attraction rather than aligning with neighbours.

The parameters of the interaction model are then inferred using MCMC methods [49]. First comparing the overall sociality of each class, we observe that calves display higher reliance on social cues, while the more mature bulls are far more autonomous and give lower weighting to near-neighbour interactions (figure 4*a*). Variation is also observed in the nature of interactions themselves. In figure 4*b* the alignment strength for adults and calves is shown. There is a clear difference here with calves showing little alignment, meaning maintaining proximity is the primary motivation. Adults, however, make greater use of the directional cues provided by neighbours.

These results are consistent with prior expectations about how different individuals are influenced by social interaction; however, they reveal that the framework we employ is able to detect and quantify this variation. This shows that our inferential framework is a means to examine how social information is used at different life stages, how this behaviour varies throughout the year, and whether persistent variation in tendencies to lead or follow [56] are found within certain individuals.

## 5. Discussion

Our study quantifies the role of social influence on fine-scale movement decisions in migrating caribou. In contrast to previous studies on other species, our results suggest that both alignment and attraction forces are important and that neither metric (interacting with all neighbours within a fixed distance) nor topological (interacting with the nearest *K* neighbours) interaction rules best represent the data. Instead a model that assigns a relative weighting to neighbours according to distance best approximates the underlying decision process. Additionally, we reveal there are strong differences in both the strength and nature of social information use between different sexes and age classes.

Although a discrete time model, the correlated random walk model is designed to cope with directional persistence [57], hence our approach is robust to the choice of time step. To ensure our results hold as the time step varies we performed our analysis using discrete time intervals of varying lengths from 1 to 10 s. We find our results are consistent over different intervals with the exponential decay model the best fitting model for each choice of interval (these results are shown in the electronic supplementary material, figure S4). Further, we examine the properties of the social vectors that result from each of our models and find there is nothing inherently more predictive about the social vectors for each model in terms of how stable they are over time, or how variable they are (see the electronic supplementary material, figure S3).

While animal social interactions are complex and cannot be represented mathematically in all but the simplest of organisms, our approach shows that models can provide insight into the key factors driving movement decisions. The modelling framework developed in McClintock *et al.* [48] combined with empirical estimation of social and environmental influence, such as those revealed in this work, have the potential to create flexible, yet rigorous, predictive models of movement for a range of taxa and environments.

The need for powerful movement and spatial use models is especially apparent for migratory species. As, by their nature, migratory populations cover a large area, the effect of a cessation of a migration has far-reaching ecological and sociological implications for the communities and ecosystems involved [1,58,59]. In the case of caribou, extensive efforts have been made to model their movement in an attempt to better understand their ecology and predict the future impacts of development and climate change [60–64]. Collective behaviour is ubiquitous in migratory populations such as these, and is thought to play a key role in driving patterns of migration and dispersal [65–68]. As movement decisions are frequently collective decisions that are influenced by the nature of social interactions and group level properties, it is essential that collective behaviour is incorporated into the modelling framework [69,70].

Ultimately, collective behaviour is important because social dynamics can have population-level implications [71]. For example, they can influence trophic interactions [72,73] and population dynamics [8] and lead to density-dependent dispersal [66]. In the context of migration, theory suggests that social travel [74] can lead to sudden collapses in migratory populations and a cessation of the migration [75,76]. Consistent with that hypothesis, the Dolphin and Union caribou studied here stopped migrating in the early 1900s when the population reached very low numbers [77]. The migration resumed in the mid-1970s once the population had increased [43,59,78], however further investigation is required in order to establish the mechanism and direction of causality underlying this link between migratory behaviour and population size.

The framework we have developed has the potential to provide much insight into the behaviour of natural populations. If embedded within longitudinal studies and combined with movement and environmental data collected at multiple scales [79], this approach will contribute to our understanding of how individual behaviour scales up to effective group-level functioning in a wide variety of taxa and ecological contexts.

## 6. Material and methods

All video processing was performed using open source freely available software packages. For computational Bayesian calculations we used the PyMC software package [49]. Further details of all methods can be found in the electronic supplementary material.

## Supplementary Material

Supporting Information

## Data accessibility

Individual caribou trajectories are available from Enlighten: Research Data (http://dx.doi.org/10.5525/gla.researchdata.579) and movebank.org (project ##416289710). Source code is available at https://github.com/ctorney/dolphinUnion.
