# Supplementary material for: Inferring the rules of social interaction in migrating caribou
Source: Philos Trans R Soc Lond B Biol Sci. 2018 Mar 26;373(1746):20170385. doi: 10.1098/rstb.2017.0385 (PMC5882989; doi:10.1098/rstb.2017.0385)
Supplement: Supporting Information [file rstb20170385supp1.pdf]

Supporting Information

Study area and species

The Dolphin and Union herd is a population of caribou (*Rangifer tarandus groenlandicus x pearyi*) that range over Victoria Island and the mainland coast of Nunavut, Canada[1]. This barren-ground caribou herd is unique as its seasonal migration to and from the wintering grounds involves a sea ice crossing between Victoria Island and the mainland[1].

UAV data collection

UAV-based filming took place in early November 2015 (Table S1) on the Western shore of Wellington Bay, Victoria Island, Canada. At this time the bulk of the caribou had not yet crossed the Dolphin and Union strait and were moving along the coast over both land and sea ice.

We approached groups of caribou to within approximately one kilometer on snowmobile and then to a distance of 100-200 meters on foot. We stayed down wind and kept terrain between us and the animals to minimize any disturbance to their behavior. We also tried to position ourselves so that the animals were moving in our direction, but not directly towards us.

When we were sufficiently close to the caribou we launched the UAV and flew to a altitude of 80 meters. We then flew horizontally until the UAV was positioned over a group of interest. Flights followed roughly the center of mass of the group as it moved and then returned to the launch site when batteries reached 30%. In some cases we flew multiple flights from the same site, but each time filmed a unique group. The UAS consisted of a 3DR Robotics Solo UAV, equipped with a Go-Pro Hero 4 camera modified with a 5.4mm no distortion lens, recording at 60fps. A Panasonic 10" rugged tablet was used as a ground station.

This work was conducted under Nunavut Wildlife Research Permit #WL2015–037 and with IACUC exemption (07/10/2015). All UAV flights were in compliance with Transport Canada regulations.

Table S1. Time and location of UAV flights

Date	Time	Latitude	Longitude
11/06/2015	15:01	69.281	-106.947
11/06/2015	15:20	69.280	-106.946
11/07/2015	11:09	69.098	-107.265
11/07/2015	11:39	69.101	-107.267
11/08/2015	11:00	69.212	-106.981
11/08/2015	12:23	69.314	-107.017
11/08/2015	12:51	69.315	-107.044

Data processing and tracking

UAV data logs

Binary log files were decoded to extract relevant coordinate and camera information. The 3DR solo employs the MAVlink protocol (`http://qgroundcontrol.org/mavlink/start`) for communication and data logging. Using the MAVlink common message set the filtered global position was extracted (lattitude, longitude, altitude calculated from multiple onboard sensors) along with the gopro heartbeat message which indicated whether the camera was recording.

In combination these data were used to associate each frame of the footage with the altitude and geographic coordinates of the UAV.

Identifying and locating caribou

All image processing was performed using the computer vision library OpenCV v3.1[2]. Identifying animals within video footage followed a two stage process. First, any objects that could potentially be a caribou were located using OpenCV's `SimpleBlobDetector` function. Next, these objects were then filtered by using a machine learning classifier. Rotational invariant features of each object were extracted according to an algorithm proposed by[3] and applied to overhead images of ungulates by[4]. These features were then passed to the classifier and the object was designated as a caribou or not. The location of each positively identified caribou, in terms of pixel coordinates within a frame, was recorded.

To assess the accuracy of caribou identification we selected 100 annotated images at random from the entire data set and manually identified caribou to compare to the performance of the algorithm. Within these 100 images there were 1630 caribou in total. Of these 1630, 1412 were correctly identified giving a true positive rate of 87%. Of the 1459 caribou that were identified by the algorithm, 1412 were indeed caribou meaning the algorithm had positive predictive value of 97%.

In order to quantify variation between different demographic groups, individual caribou were manually assigned to one of the following classes, large bull, adult (small bulls, cows and yearlings), and calf.

Correcting for camera movement

As the camera was constantly in motion during the filming of caribou herds, the positions of individuals within the image had to be transformed to a fixed coordinate system. This was achieved by calculating the motion of the camera from the movement across the frame of landscape features. Each frame was first preprocessed using the `equalizeHist` function within OpenCV to improve image contrast (see Fig. S2). The motion between successive frames of the video was then calculated using the `findTransformECC` function. The cumulated coordinate transformation for each frame was then found by iteratively applying each successive camera movement to the position data,

$$\begin{bmatrix} x' \\ y' \\ 1 \end{bmatrix} = \mathbf{A}_1 \dots \mathbf{A}_{t-1}\mathbf{A}_t \begin{bmatrix} x \\ y \\ 1 \end{bmatrix} \tag{S1}$$

where $\mathbf{A}_t$ is the geometric transform matrix that maps frame t to frame $t-1$, $[x,y]$ are the pixel coordinates of the individual in the image frame, and $[x',y']$ are the coordinates relative to a fixed reference frame.

Track linking and smoothing.

Once individuals were located within single frames in a fixed coordinate system, trajectories were created by linking individuals over time using the trackpy library[5]. Trajectories were then smoothed using a Kalman filter.

Movement model

The movement process model is a discrete time, continuous space correlated random walk[6]. Movement trajectories are discretized into 2-second intervals with the step defined by the vector $\mathbf{V}_t$ and the step direction $\theta = \text{atan}(\mathbf{V}^y, \mathbf{V}^x)$ defined relative to a fixed orientation. Movement steps are biased toward social and environmental cues and the strength of these biases are inferred using Bayesian computational methods. Markov chain Monte Carlo methods were used within the package `pymc`[7] to find the posterior probability distribution of parameters conditional on the observed movement data. A single chain of 400000 iterations with a burn-in period of 50000 iterations was used for all models. Summary statistics and maximum likelihood estimates of parameter values for all models are shown in Table S4.

Following[6], each movement step is modeled as a random draw from a wrapped Cauchy distribution centered on an expected heading. The probability the movement step at time t is in the direction θ_t is given by

$$P(\theta_t|\rho,\boldsymbol{\alpha}) = \frac{1}{2\pi}\frac{1-\rho^2}{1+\rho^2-2\rho\cos\left(\theta_t-\lambda_t(\boldsymbol{\alpha})\right)}. \tag{S2}$$

where λ_t is the expected heading defined as a function of model parameters $\boldsymbol{\alpha}$, and ρ determines the variance around this heading. Various drivers of movement may be incorporated into the model by taking λ_t to be a weighted average of the heading indicated by each potential influence. In total we compare eight models that incorporate three potential drivers of movement decisions: directional persistence, environmental features, and social cues. The expected heading used within (S2) is a weighted average of the headings associated with each of these factors,

$$\lambda_t = \text{atan2}\left(\alpha\sin\psi_t + \beta\sin\phi_t + \gamma\sin\theta_{t-1}, \alpha\cos\psi_t + \beta\cos\phi_t + \gamma\cos\theta_{t-1}\right) \tag{S3}$$

where $\alpha+\beta+\gamma=1$, ψ_t is the heading dictated by social cues, ϕ_t is an estimate of environmental forces, and θ_{t-1} is the previous heading.

Correlated random walk model

The first model ignores all environmental and social cues and assumes individuals move according to a correlated random walk with step directions drawn from a wrapped Cauchy distribution centered on the current heading. The probability density function for direction θ at time t is defined by

$$P(\theta_t|\rho) = \frac{1}{2\pi}\frac{1-\rho^2}{1+\rho^2-2\rho\cos\left(\theta_t-\theta_{t-1}\right)}. \tag{S4}$$

Here ρ determines the strength of persistence in movement. A value of $\rho=0$ means a direction is selected with uniform probability from the interval $[0,2\pi]$ at every time step, while if $\rho=1$ then the heading is effectively fixed. The posterior distribution $\pi(\rho|\boldsymbol{\theta})$ can be found, subject to a normalizing constant, by application of Bayes' rule

$$\pi(\rho|\boldsymbol{\theta}) \propto P(\boldsymbol{\theta}|\rho)\pi(\rho) \tag{S5}$$

where $\boldsymbol{\theta}$ is the vector of all movements and $\pi(\rho)$ is a flat prior distribution uniform on the interval $[0,1]$ (prior distributions for parameters of all models can be found in Table S4).

Correlated random walk with environmental influence

Following the approach of[8] we approximate the effect of environmental features in the movement model by finding the average heading of all individuals at every fixed point in space. For each individual i at position $\mathbf{x}$ we calculate an estimated environmental vector by taking a weighted average of the heading of all other individuals at all times, weighted according to a Gaussian distance-decay function.

$$\phi = \text{atan}\left(\sum_{j=0,j\neq i}^{N} \omega(d_{ij}) v^{y}{}_{j}, \sum_{j=0,j\neq i}^{N} \omega(d_{ij}) v^{x}{}_{j}\right) \tag{S6}$$

where the summation is taken over all other individuals at all times, $\mathbf{V}_j$ is the movement step of individual j, d_{ij} is the distance between i and j and

$$\omega(y) = \exp\left(-\frac{y^2}{\kappa^2}\right). \tag{S7}$$

The probability density function for the movement direction is then dependent on the previous heading and the environmental vector. We introduce the angle λ which is a weighted combination of the current heading and the environment as defined by Eqn. S6,

$$\lambda_t = \text{atan2}\left(\beta \sin\phi_t + (1-\beta)\sin\theta_{t-1}, \beta\cos\phi_t + (1-\beta)\cos\theta_{t-1}\right) \tag{S8}$$

This is now the expected heading and

$$P(\theta_t|\rho,\beta) = \frac{1}{2\pi}\frac{1-\rho^2}{1+\rho^2-2\rho\cos\left(\theta_t - \lambda_t(\beta)\right)}. \tag{S9}$$

The posterior distribution may now be written as

$$\pi(\rho,\beta|\boldsymbol{\theta}) \propto P(\boldsymbol{\theta}|\rho,\beta)\pi(\rho)\pi(\beta) \tag{S10}$$

Socially-influenced movement model

Using the same framework we may include the effects of social interactions on movement decisions by incorporating a component into Eqn. S8 to create a socially-informed model[9]. Introducing a social angle ψ_t which resolves all social cues into a single heading, we may write the expected heading for a movement step as

$$\lambda_t = \text{atan2}\left(\alpha\sin\psi_t + \beta\sin\phi_t + \gamma\sin\theta_{t-1}, \alpha\cos\psi_t + \beta\cos\phi_t + \gamma\cos\theta_{t-1}\right) \tag{S11}$$

where $\alpha+\beta+\gamma = 1$ and their values dictate the relative weightings given to each source of influence.

The social angle ψ_t is calculated by taking a weighted average of a combination of the angle toward a neighbour and the heading of that neighbour. By altering the functional form of the weighting different social models may be compared. We evaluate the performance of three forms of interactions, a metric interaction zone where individuals are influenced equally by all neighbours within a fixed range, a topological interaction model where at most the nearest K neighbours affect decisions (if there are fewer than K individuals in the video frame, then the interactions are limited to all those in the frame), and a model where influence decays exponentially with distance. All sets of interaction rules are assessed with and without alignment forces, meaning a total of 6 socially-informed movement models are compared.

The resulting social heading for individual i, denoted $\psi_t(i)$ is defined as a sum of the influence of each neighbour, weighted according to the relative distance and orientation of the neighbour. Both the relative position of individual j, denoted by the vector $\mathbf{r}_{ij}$ and the heading of the neighbour, $\theta_{t-1}(j)$, contribute to the resultant social vector, hence

$$\psi_t(i) = \text{atan2}\left(\sum_{j\neq i}\omega(\mathbf{r}_{ij})\left(\frac{r^y_{ij}}{|\mathbf{r}_{ij}|} + \eta\sin\theta_{t-1}(j)\right), \sum_{j\neq i}\omega(\mathbf{r}_{ij})\left(\frac{r^x_{ij}}{|\mathbf{r}_{ij}|} + \eta\cos\theta_{t-1}(j)\right)\right) \tag{S12}$$

where η controls the strength of alignment forces, and $\omega(.)$ is a function of the relative position vector that defines the nature of the social interaction rules.

For the metric interaction rules the weighting function is defined as

$$\omega(\mathbf{r}_{ij}) = \begin{cases} 1 & \text{if } -V/2 < \angle\mathbf{r}_{ij} < V/2 \text{ and } D_N < |\mathbf{r}_{ij}| < D_I \\ 0 & \text{otherwise} \end{cases} \tag{S13}$$

where $V \in [-\pi, \pi]$ is the interaction angle, D_N is a neutral distance where neighbours within this range are excluded as being too close, and D_I the maximum distance at which caribou interact. Similarly the weighting function for topological interactions may be written as

$$\omega(\mathbf{r}_{ij}) = \begin{cases} 1 & \text{if } -V/2 < \angle \mathbf{r}_{ij} < V/2, j < K \text{ and } |\mathbf{r}_{ij}| > D_N \\ 0 & \text{otherwise} \end{cases} \tag{S14}$$

where K represents the maximum number of neighbours in the interaction range and neighbours are counted in order of distance from the focal individual. Finally the exponentially decaying interaction range is obtained using the following function,

$$\omega(\mathbf{r}_{ij}) = \begin{cases} \exp\left(-\frac{|\mathbf{r}_{ij}|}{D_I}^{\delta}\right) & \text{if } -V/2 < \angle \mathbf{r}_{ij} < V/2 \text{ and } |\mathbf{r}_{ij}| > D_N \\ 0 & \text{otherwise} \end{cases} \tag{S15}$$

where D_I controls the interaction length scale and δ the rate of decay of influence beyond this scale.

The likelihood for the metric interaction model can be written as

$$P(\theta_t|\rho, \alpha, \beta, \gamma, D_N, D_I, V) = \frac{1}{2\pi} \frac{1-\rho^2}{1+\rho^2 - 2\rho\cos\left(\theta_t - \lambda_t(\alpha, \beta, \gamma, D_N, D_I, V)\right)}. \tag{S16}$$

where D_N is the neutral zone distance, D_I is the interaction distance, and V is the interaction angle. The posterior distribution of the parameters is then

$$\pi(\rho, \alpha, \beta, \gamma, D_N, D_I, V|\boldsymbol{\theta}) \propto P(\boldsymbol{\theta}|\rho, \alpha, \beta, \gamma, D_N, D_I, V)\pi(\rho)\pi(\alpha)\pi(\beta)\pi(\gamma)\pi(D_N)\pi(D_I)\pi(V). \tag{S17}$$

All social interaction models follow the same form with different parameter sets controlling the social angle ψ. Prior distributions for all parameters are shown in Table S4.

Validation of methodology with simulated data

To validate the method we employ here, we apply the same methods to synthetic data and compare the results to the known parameters of the simulations. We simulate 5 scenarios, asocial individuals performing a random walk, asocial individuals following an external cue, asocial individuals following a trail, individuals interacting via a metric interaction zone, and individuals interacting via a topological interaction distance. To ensure we evaluate the discrete-time approximation involved in our method, a 2-second inferred movement step corresponds to 20 simulated timesteps.

Correlated random walk simulation

We simulated 20 individuals moving in an unbounded domain with the following update rule

$$\begin{array}{ccc} x_i(t+\Delta t) & & x_i(t) + \Delta t \cos\theta_i(t) \\ y_i(t+\Delta t) & = & y_i(t) + \Delta t \sin\theta_i(t) \\ \theta_i(t+\Delta t) & & \theta_i(t) + N(0, \sigma^2 \Delta t) \end{array} \tag{S18}$$

We simulated this model for 4000 timesteps, resetting all individuals to the centre of the domain every 500 timesteps to restrict dispersal, then applied our inference framework to compare the performance of the correlated random walk model, the environmental model and the social models with alignment. ΔWAIC scores are shown in Table S2, indicating the random walk model was correctly selected. Note, WAIC attributes a penalty of zero to a parameter if the learning comes entirely from the prior.

External cue simulation

We next incorporated an external biased movement into the simulation. We simulated 100 individuals for 2000 timesteps with the following update rule

$$\begin{array}{ccc} x_i(t+\Delta t) & & x_i(t) + \Delta t \cos\theta_i(t) \\ y_i(t+\Delta t) & = & y_i(t) + \Delta t \sin\theta_i(t) \\ \theta_i(t+\Delta t) & & \theta_i(t) + \nu(\bar{\theta} - \theta_i(t))\Delta t + \sigma dW_t \end{array} \tag{S19}$$

where ν is a mean reversion parameter, $\bar{\theta}$ is a global average heading and dW_t is a standard Wiener process. We set $\bar{\theta} = 0$, hence the orientation angle of individuals follows an Ornstein-Uhlenbeck process with average heading directed along the x-axis. Model fitting was performed as with the empirical data and ΔWAIC scores are shown in Table S2.

Table S2. Model selection scores for individual-based non-social simulations.

Model	CRW ΔWAIC	External Bias ΔWAIC	Trail Following ΔWAIC
Random walk	0	0	0
Environment	0	-2462	-8414
Metric + alignment	2	-2460	-8414
Exponential decay + alignment	2	-2460	-8414
Topological + alignment	2	-2462	-8414

Trail following simulation

To assess our methods ability to detect independent individuals travelling along a trail, we simulated 10 individuals following a randomly generated stochastic path. To generate a trail we used an Ornstein-Uhlenbeck process and set the trail location to be the value of this process. Individuals within the simulation moved toward the trail if they were either side of it, then along the trail once they were sufficiently close to it. Snapshots from a simulation of this process are shown in Fig. S1. We simulated 10 individuals moving along the trail for 5000 timesteps then performed 10 independent replications, randomly locating the individuals at the start of a newly generated trail for each replicate.

Each of our movement models was then applied to the data and model fit was compared using WAIC. Scores are shown in Table S2. Results showed that the environmental model was a better fit than the random walk model, while including social interactions did not improve model scores.

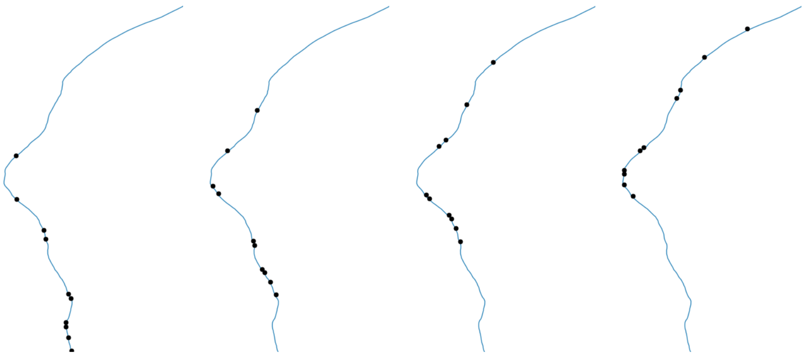

Figure S1. Simulation of non-social individuals following the same trail. Panels show different snapshots from the same simulation.

Inference of simulated interaction rules

To compare our methods ability to differentiate between different interaction rules, we performed individual-based simulations using metric and topological interactions. We simulated 100 individuals moving in an unbounded domain for 2000 timesteps. Individuals updated their states according to

$$\begin{matrix} x_i(t+\Delta t) \\ y_i(t+\Delta t) \\ \theta_i(t+\Delta t) \end{matrix} = \begin{matrix} x_i(t)+\Delta t\cos\theta_i(t) \\ y_i(t)+\Delta t\sin\theta_i(t) \\ \theta_i(t)+\nu(\bar{\psi}_i-\theta_i(t))\Delta t+\sigma dW_t \end{matrix} \tag{S20}$$

where $\bar{\psi}$ indicates the heading derived from social interactions. The social vector was defined by the following rules. Firstly if a neighbour is located within the repulsion zone of the focal individual then,

$$\psi_i = \text{atan2}\left(-\sum_{j\neq i} r^y_{ij}, -\sum_{j\neq i} r^x_{ij}\right) \tag{S21}$$

where the summation is taken over all individuals within the repulsion zone. Note, this is different to the assumption of the movement model in that active movement away from near-neighbours occurs.

If no neighbour is within the repulsion zone the focal individual then switches to attraction and alignment rules, so that

$$\psi_i = \text{atan2}\left(\sum_{j\neq i}\omega(\mathbf{r}_{ij})\left(\frac{r^y_{ij}}{|\mathbf{r}_{ij}|}+\eta\sin\theta_j(t)\right), \sum_{j\neq i}\omega(\mathbf{r}_{ij})\left(\frac{r^x_{ij}}{|\mathbf{r}_{ij}|}+\eta\cos\theta_j(t)\right)\right) \tag{S22}$$

For the metric interaction rules the weighting function is defined as

$$\omega(\mathbf{r}_{ij}) = \begin{cases} 1 & \text{if } -V/2 < \angle\mathbf{r}_{ij} < V/2 \text{ and } D_R < |\mathbf{r}_{ij}| < D_I \\ 0 & \text{otherwise} \end{cases} \tag{S23}$$

where $V \in [-\pi, \pi]$ is the interaction (visual) angle, D_R is the repulsion distance, and D_I the interaction range. For the simulation of topological interactions the weighting is

$$\omega(\mathbf{r}_{ij}) = \begin{cases} 1 & \text{if } -V/2 < \angle\mathbf{r}_{ij} < V/2, j < K \text{ and } |\mathbf{r}_{ij}| > D_R \\ 0 & \text{otherwise} \end{cases} \tag{S24}$$

where K represents the maximum number of neighbours in the interaction range and neighbours are counted in order of distance from the focal individual.

We took the trajectories from both simulations and applied the discrete-time framework to select the best model and infer model parameters. ΔWAIC scores compared to the random walk probability model are shown in Table S3. These results reveal that in both cases the lowest WAIC score, and thus the best supported model, corresponds to the correct simulation model. We note that overall the topological simulation is more strongly identified and this is because the metric model reverts to a random walk when no neighbours are within an individual's interaction zone. This means the random walk model (the baseline WAIC score) performs comparatively better for the metric simulation than for the topological model.

We further compare the inferred parameters of the best-fitting model with the simulation parameters employed and a comparison is shown in Fig S5. Again we find good agreement between output from the inference procedure and known properties of the simulations.

Table S3. Model selection scores for individual-based social simulations.

Model	Metric Simulation ΔWAIC	Topological Simulation ΔWAIC
Metric + alignment	-1198	-1212
Exponential decay + alignment	-1195	-1441
Topological + alignment	-1093	-1449

Table S4. Posterior distributions, inferred values and maximum likelihood estimates of parameters for movement models. U(a,b) indicates a continuous uniform distribution on the interval (a,b), U{a,b} is a discrete uniform distribution.

Model	Parameter	Units	Prior distribution	Posterior mean	95% CI	MLE
CRW	Cauchy dispersion (ρ)	-	U(0,1)	0.9324	[0.9312,0.9337]	0.9324
CRW with environment	Cauchy dispersion (ρ)	-	U(0,1)	0.9357	[0.9345,0.9368]	0.9357
	Environment weighting (β)	-	U(0,1)	0.1574	[0.1498,0.1651]	0.1574
Metric interaction	Cauchy dispersion (ρ)	-	U(0,1)	0.9394	[0.9383,0.9406]	0.9395
	Social weighting (α)	-	U(0,1)	0.3568	[0.3388,0.3748]	0.3572
	Environment weighting (β)	-	U(0,1)	0.0903	[0.0842,0.0964]	0.0901
	Persistence weighting (γ)	-	U(0,1)	0.5529	[0.5373,0.5684]	0.5527
	Neutral zone length (D_N)	meters	U(0,2)	1.32	[1.23,1.37]	1.31
	Interaction zone (D_I)	meters	U(2,20)	10.15	[10.12,10.19]	10.14
	Interaction angle (V)	radians	U(0,2π)	0.503	[0.501,0.504]	0.504
Metric with alignment	Cauchy dispersion (ρ)	-	U(0,1)	0.9395	[0.9383,0.9405]	0.9395
	Social weighting (α)	-	U(0,1)	0.3712	[0.3531,0.3893]	0.3722
	Environment weighting (β)	-	U(0,1)	0.0859	[0.0800,0.0920]	0.0857
	Persistence weighting (γ)	-	U(0,1)	0.5429	[0.5273,0.5586]	0.5421
	Neutral zone length (D_N)	meters	U(0,5)	1.30	[1.22,1.37]	1.31
	Interaction zone (D_I)	meters	U(0.5,20)	10.13	[9.85,10.19]	10.13
	Interaction angle (V)	radians	U(0,2π)	0.503	[0.501,0.505]	0.504
	Alignment weight (η)	-	U(0,1)	0.57	[0.406,0.744]	0.56
Exponential decay	Cauchy dispersion (ρ)	-	U(0,1)	0.9395	[0.9383,0.9406]	0.9395
	Social weighting (α)	-	U(0,1)	0.3508	[0.3328,0.3689]	0.3516
	Environment weighting (β)	-	U(0,1)	0.0920	[0.0858,0.0982]	0.0920
	Persistence weighting (γ)	-	U(0,1)	0.5572	[0.5418,0.5727]	0.5564
	Neutral zone length (D_N)	meters	U(0,5)	1.33	[1.25,1.38]	1.29
	Decay length (D_I)	meters	U(0.5,20)	7.98	[5.77,8.58]	7.97
	Decay exponent (δ)	-	U(1,25)	19.01	[8.13,24.65]	19.31
	Interaction angle (V)	radians	U(0,2π)	0.503	[0.501,0.503]	0.504
Exponential decay with alignment	Cauchy dispersion (ρ)	-	U(0,1)	0.9396	[0.9385,0.9408]	0.9397
	Social weighting (α)	-	U(0,1)	0.3839	[0.3642,0.4030]	0.3839
	Environment weighting (β)	-	U(0,1)	0.0843	[0.0784,0.0903]	0.0843
	Persistence weighting (γ)	-	U(0,1)	0.5318	[0.5152,0.5489]	0.5318
	Neutral zone length (D_N)	meters	U(0,5)	1.33	[1.25,1.38]	1.30
	Decay length (D_I)	meters	U(0.5,20)	5.01	[4.44,5.54]	5.07
	Decay exponent (δ)	-	U(1,25)	9.17	[7.30,11.45]	9.23
	Interaction angle (V)	radians	U(0,2π)	0.552	[0.550,0.561]	0.553
	Alignment weight (η)	-	U(0,1)	0.77	[0.618,0.945]	0.76
Topological	Cauchy dispersion (ρ)	-	U(0,1)	0.9386	[0.9375,0.9398]	0.9386
	Social weighting (α)	-	U(0,1)	0.2188	[0.2060,0.2316]	0.2202
	Environment weighting (β)	-	U(0,1)	0.1182	[0.1114,0.1251]	0.1178
	Persistence weighting (γ)	-	U(0,1)	0.6630	[0.6507,0.6754]	0.6620
	Neutral zone length (D_N)	meters	U(0,5)	1.29	[1.16,1.37]	1.30
	neighbours interacted with (K)	-	U{0,20}	1	-	1
	Interaction angle (V)	radians	U(0,2π)	0.563	[0.554,0.569]	0.562
Topological with alignment	Cauchy dispersion (ρ)	-	U(0,1)	0.9387	[0.9376,0.9398]	0.9387
	Social weighting (α)	-	U(0,1)	0.2358	[0.2229,0.2488]	0.2357
	Environment weighting (β)	-	U(0,1)	0.1102	[0.1034,0.1171]	0.1103
	Persistence weighting (γ)	-	U(0,1)	0.6540	[0.6418,0.6662]	0.6540
	Neutral zone length (D_N)	meters	U(0,5)	1.29	[1.08,1.37]	1.32
	neighbours interacted with (K)	-	U{0,20}	1	-	1
	Interaction angle (V)	radians	U(0,2π)	0.567	[0.562,0.569]	0.569
	Alignment weight (η)	-	U(0,2)	1.00	[0.7561,1.2499]	1.00

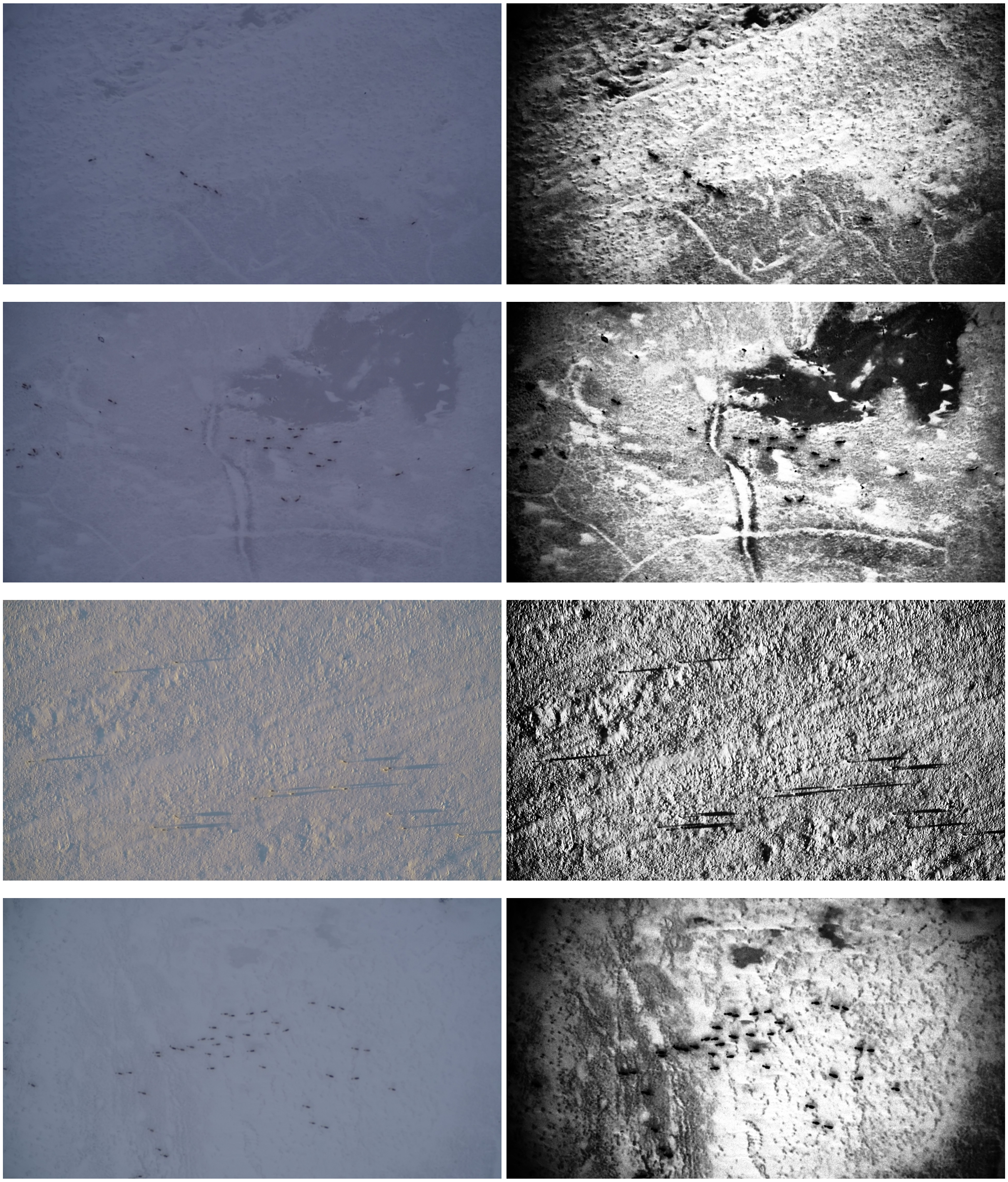

Figure S2. Quantifying camera motion. In order to separate camera motion from animal motion, frames were aligned using an area-based algorithm that detected similarities in intensity. Frames were pre-processed to enhance contrast and normalize brightness. A random set of four images are shown here to illustrate how, even in relatively sparse landscapes, video frames contained sufficient background structure for camera motion detection.

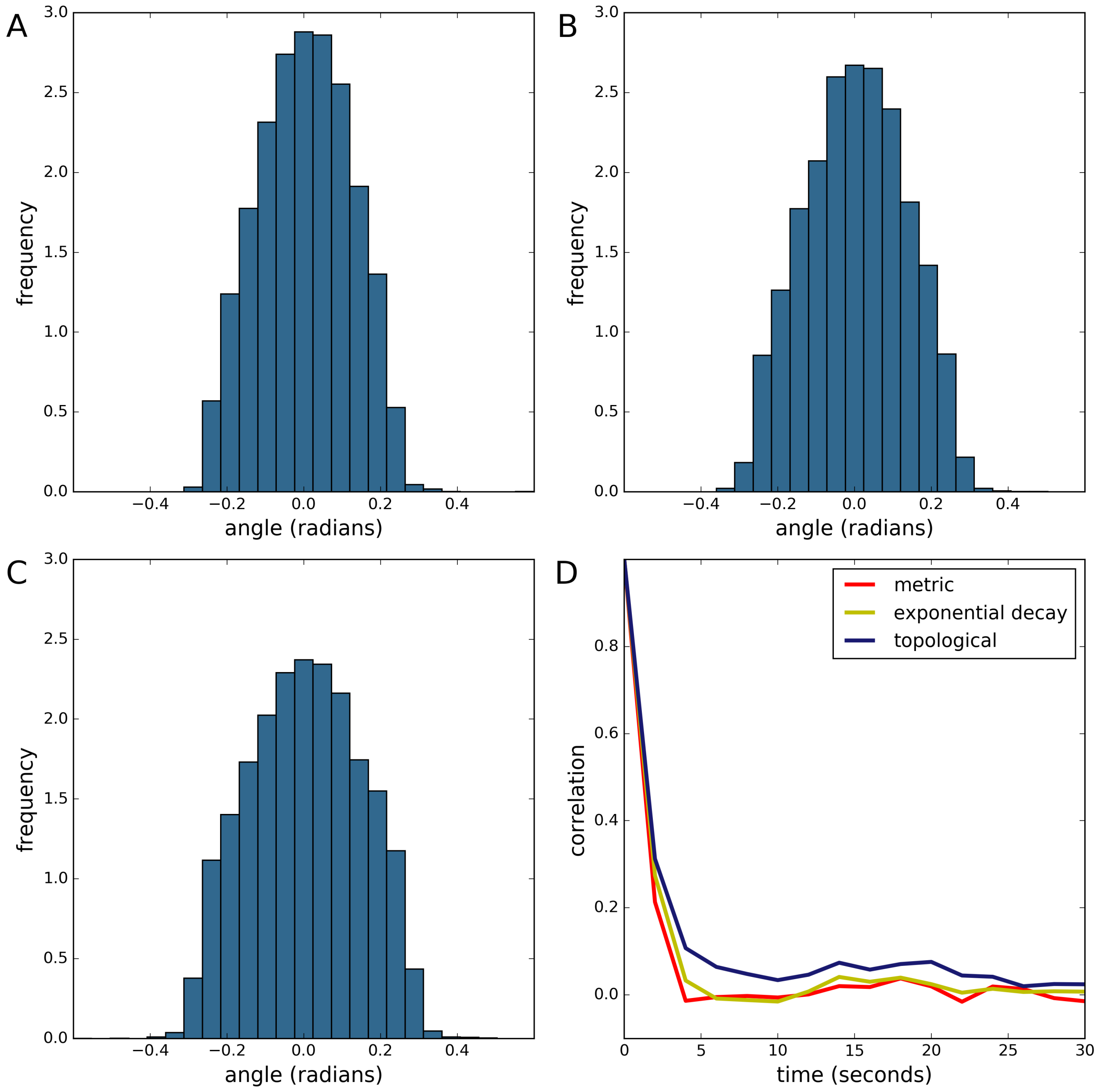

Figure S3. Comparison of social interaction vector resulting from the different models. (A-C) Histogram of headings indicated by social interation rules corresponding to the metric model (A), the decaying exponential model (B) and the topological model (C). (D) Autocorrelation of the modelled social vector. All models display similar properties in terms of the distribution of angles that results and the autocorrelation, or stability, of the angle.

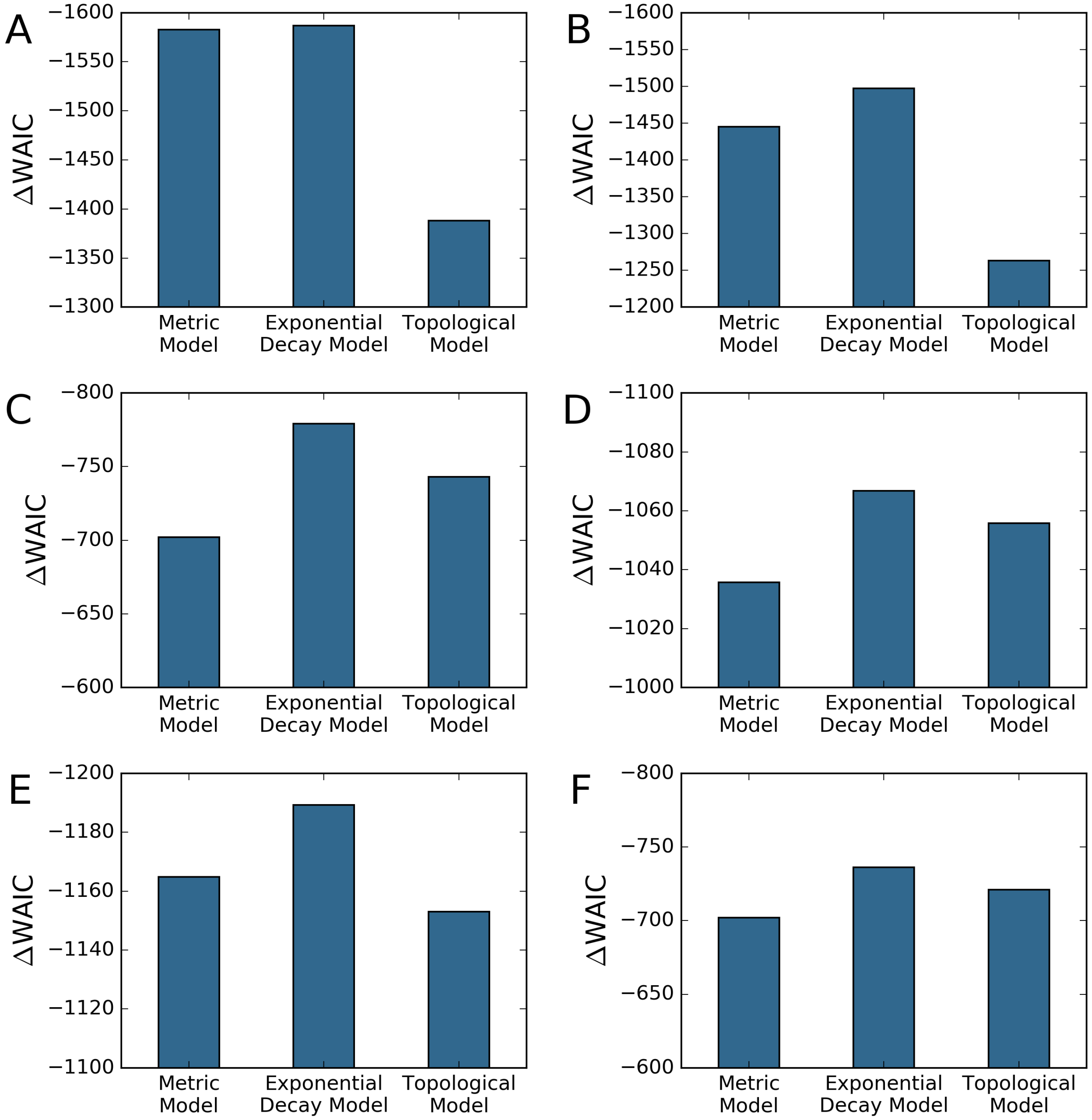

Figure S4. Model comparison for different values of discrete time-step. To ensure results are robust to the choice of time step models were tested using data based on calculating move steps using (A) 1-second interval, (B) 2-second interval (as in main text), (C) 4-second interval, (D) 6-second interval, (E) 8-second interval, (F) 10-second interval. WAIC values are reported compared to the environmental model score for the corresponding interval. Note, the number of data points changes as the time step changes so values are not directly comparable across time intervals.

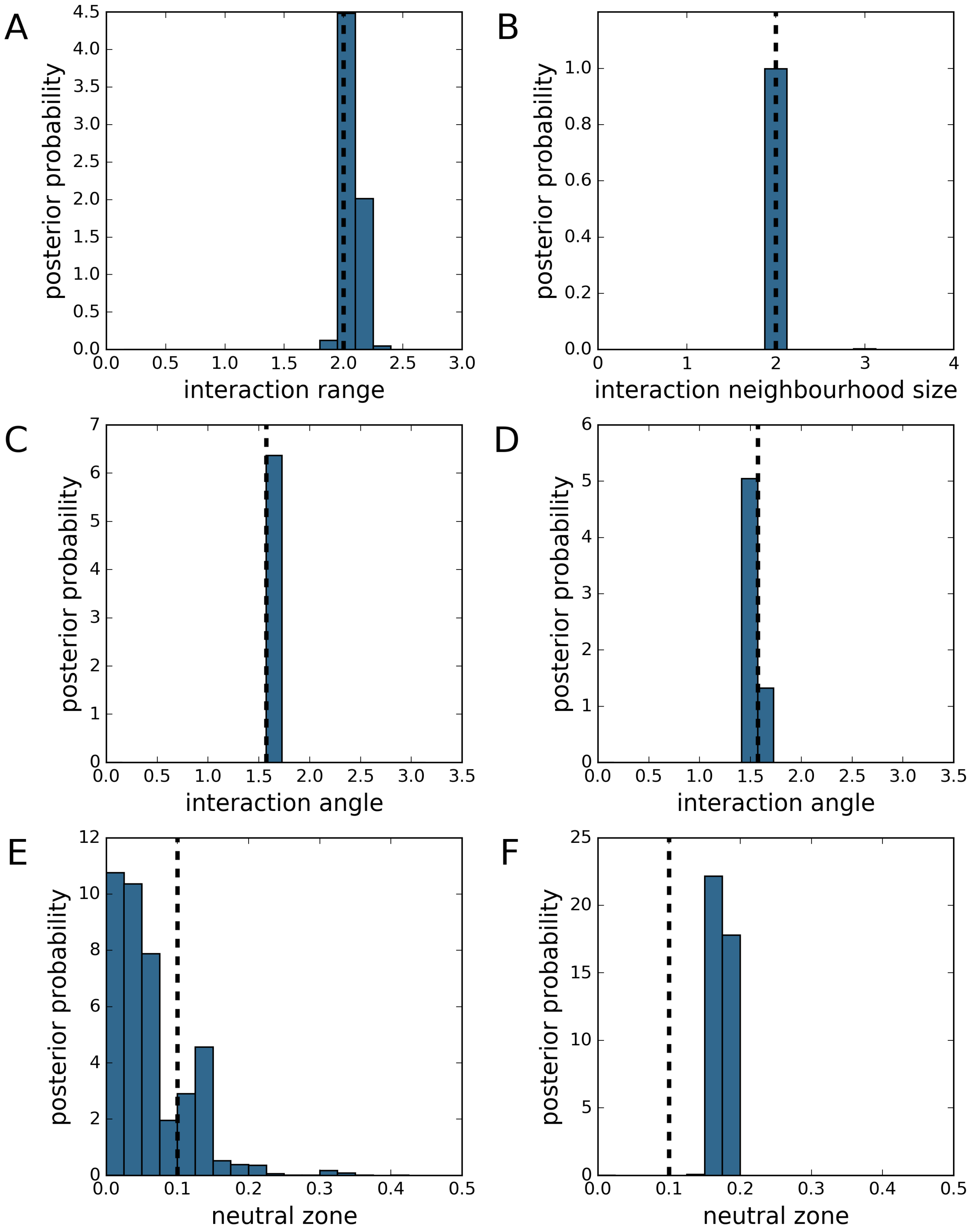

Figure S5. Validation on simulated data. The model selection framework was applied to simulations of a metric interaction model (A,C,E) and a topological interaction model (B,D,F). Panels show posterior distributions from MCMC runs for the interaction range (A and B), the interaction angle (C and D), and the neutral zone (E and F). Simulation parameters are indicated by the dashed line. Inferred and simulated parameters show good agreement except for the neutral zone. This is likely due to the difference between model assumptions (individuals within this zone are ignored) and the simulation rules (the focal individual turns away from neighbours in this zone).

References

1. Dumond, M. & Lee, D. S. Dolphin and union caribou herd status and trend. *Arctic* 329–337 (2013).
2. Bradski, G. *Dr. Dobb's Journal of Software Tools* (2000).
3. Liu, K. *et al.* Rotation-invariant hog descriptors using fourier analysis in polar and spherical coordinates. *International Journal of Computer Vision* **106**, 342–364 (2014).
4. Torney, C. J. *et al.* Assessing rotation-invariant feature classification for automated wildebeest population counts. *PloS one* **11**, e0156342 (2016).
5. Allan, D., Caswell, T., Keim, N. & van der Wel, C. trackpy: Trackpy v0.3.0 (2015). URL `http://dx.doi.org/10.5281/zenodo.34028`.
6. McClintock, B. T. *et al.* A general discrete-time modeling framework for animal movement using multistate random walks. *Ecological Monographs* **82**, 335–349 (2012).
7. Patil, A., Huard, D. & Fonnesbeck, C. J. Pymc: Bayesian stochastic modelling in python. *Journal of statistical software* **35**, 1 (2010).
8. Dalziel, B. D., Corre, M. L., Côté, S. D. & Ellner, S. P. Detecting collective behaviour in animal relocation data, with application to migrating caribou. *Methods in Ecology and Evolution* **7**, 30–41 (2016).
9. Haydon, D. T. *et al.* Socially informed random walks: incorporating group dynamics into models of population spread and growth. *Proceedings of the Royal Society of London B: Biological Sciences* **275**, 1101–1109 (2008).
